# Recommendations for Patients with High Return to Sports Expectations after TKA Remain Controversial

**DOI:** 10.3390/jcm10010054

**Published:** 2020-12-26

**Authors:** Tu-Lan Vu-Han, Clemens Gwinner, Carsten Perka, Sebastian Hardt

**Affiliations:** Department of Orthopaedic Surgery and Traumatology, Charité Berlin, University Hospital, Chariteplatz 1, 10117 Berlin, Germany; clemens.gwinner@charite.de (C.G.); carsten.perka@charite.de (C.P.); sebastian.hardt@charite.de (S.H.)

**Keywords:** total knee arthroplasty, return to sports, joint replacement, physical activity, TKA design

## Abstract

(1) Background: Improved surgical techniques and implants in total knee arthroplasty (TKA) have led to broader indications for surgical interventions of osteoarthritis of the knee. There is a growing young and active patient subgroup with high return to sports (RTS) expectations after TKA. The current lack of evidence regarding RTS capacity in this patient cohort, requires the consolidation of experts’ opinions and experiences to address the special needs among these patients. The aim of this study was to assess current expert opinions in regard to preoperative patient assessment, surgical technique and decision-making and patient counseling for these patients. (2) Methods: We performed a survey among surgeons specialized in arthroplasty with a questionnaire designed to assess current recommendations, surgical techniques, and implant preferences as well as patient counseling in patients with high expectations for RTS after TKA. (3) Results: The majority of surgeons are in favor of return to low-impact sports after TKA within 3 to 6 months. Some even recommend return to high-impact sports. Despite improvement of surgical techniques and implants, we observed no clear preference for a single surgical technique or implant specification in active patients. (4) Conclusions: Current evidence for sports-associated complications after TKA is scarce. Despite a growing array of surgical techniques and implants, the available literature is still controversial with no single surgical technique or TKA design distinguishing itself clearly from others. Surgeons’ recommendations are mostly based on their experience and training. Nonetheless, we observed growing faith in modern implants with some surgeons even recommending high-impact sports after TKA.

## 1. Introduction

The benefits of an active lifestyle to prevent chronic diseases, such as cardiovascular disease, obesity, type 2 diabetes mellitus, hypertension, osteoporosis, and even depression have been demonstrated and emphasized extensively in the current literature [1,2]. Meanwhile, an aging population as well as obesity epidemic are inciting a rising incidence of osteoarthritis of the knee. In order to maintain or return to an active lifestyle, surgical intervention for joint arthroplasty is often indicated. In addition, patients suffering from osteoarthritis of the knee would likely benefit from counselling to encourage an active lifestyle after knee arthroplasty, as several publications have observed an increased incidence of preconditions such as cardiovascular disease, obesity, diabetes mellitus, and hypertension in this patient cohort [3,4,5].

Alongside these developments, knee arthroplasty surgical techniques and implant designs have improved tremendously since the first vitallium prostheses attempts began in 1940s by Campbell, Walldius, Rocher, Kraft, Levinthal, and others [6]. Knee arthroplasties have become a standard medical intervention with current prediction models reporting an expected rise of up to 401% for TKAs within the next 20 years [7,8]. Current mid- and long-term outcomes show up to 80% postoperative patient satisfaction [9,10]. Surgical indication has thus expanded toward a more active and often younger patient cliental (<65 years of age) with high return to sports (RTS) expectations [11,12,13,14]. Improving outcomes for the remaining 20% is an ongoing endeavor. 

Studies have shown that patient satisfaction is strongly dependent on the return to physical activity after surgical intervention. The identified main drivers for patient satisfaction include pain, physical health, and mental health [9,15,16,17,18]. In turn, patients’ level of postoperative activity is likely influenced by their physicians’ recommendations and support [19]. However, to this date no uniform recommendations regarding return to sports after knee arthroplasty exist. The current literature remains controversial regarding the RTS recommendations for patients after knee arthroplasty [20].

We conducted a survey among German arthroplasty experts to assess their patient-assessment, surgical decisions in regard to surgical approach, choice of implant design and materials as well as patient counseling in patients with high RTS expectations. These patients are likely a growing subgroup among TKA patients. The questionnaire was designed for surgeons specialized in joint arthroplasty. The aim was to identify, whether surgeons make specific decisions to address the heightened expectations in this specific subgroup. 

## 2. Experimental Section

We developed a questionnaire in close dialogue with the leadership of the German Arthroplasty Society (AE—Arbeitsgemeinschaft für Endoprothetik)—Germany’s largest and leading society of hip and knee arthroplasty surgeons. For the development of the questionnaire a committee was formed that developed a first draft, which was then shared for feedback and comments with leading surgeons in the field. The feedback was then incorporated for the final version of the questionnaire. The questions were designed to evaluate current recommendations regarding return to sports after total knee arthroplasty (TKA). We assessed what surgeons currently recommend, what implant specifications and surgical techniques they prefer in patients with high RTS expectations and what factors may influence some of their decision-making. 

We distributed 300 questionnaires among surgeons specialized in hip and knee arthroplasty and members of the AE, who attended the 21st Annual AE Conference in December 2019. In total, 344 participants were registered for the meeting. Membership approval in the AE society requires a completed residency in orthopedic and trauma surgery with sub-specialization in arthroplasty and an endorsement by an AE member. AE members must perform at least 50 arthroplasty surgeries per annum to maintain status. Of the 300 distributed questionnaires, 101 were returned, equaling a 34% response rate.

The original questionnaire was designed in German. The original, as well as an English translation of the questionnaire, are provided in the Supplementary S1 and S2. The questionnaire was conceptualized and designed to contain 5 general questions and 15 specific items to knee arthroplasty including 35 sub-items. When multiple choices were possible, absolute counts are displayed in the figures. The questionnaire assessed preoperative patient factors that influence implant longevity and surgical decision-making, such as surgical planning, surgical approach, implant design, postoperative treatment, and counseling in patients with high return to sports (RTS) expectations.

Previous surveys performed among American surgeons used the Clifford and Mallon classification to distinguish between low- and high-impact sports [21]. These classifications are not based on in vivo measured biomechanical data for each specific type of sport. For the purpose of our questionnaire, we characterized low-impact sports as smooth and gentle in movements. In contrast, high-impact sports were characterized by rapid and abrupt movements, with heightened risk of injury, especially without prior training.

For data analysis, each returned questionnaire received a unique identity (UID) and answers given were coded into an R data frame for statistical analysis. The data frame is provided in the Supplementary S3. The returned questionnaires were analyzed using R Version 3.6.3 by the R Foundation for Statistical Computing and figures were produced using the package ggplot2 [22]. This study did not require ethical approval as no human subjects were involved and the participation in the survey was voluntary.

## 3. Results

### 3.1. Survey Participants

Our survey was conducted among surgeons specialized in knee and hip arthroplasty. Of 300 distributed questionnaires 101 were returned, corresponding with a response rate of 34%. In total, 86.13% of survey participants had more than ten years of surgical experience, and 55.44% more than twenty years of surgical experience; 54.46% stated a high (i.e., performing sports multiple times a week) and 38.61% a medium (i.e., occasional participation in sports) level of personal physical activity.

### 3.2. Perioperative Assessments Regarding Return to Sports after TKA

**Figure 1:**
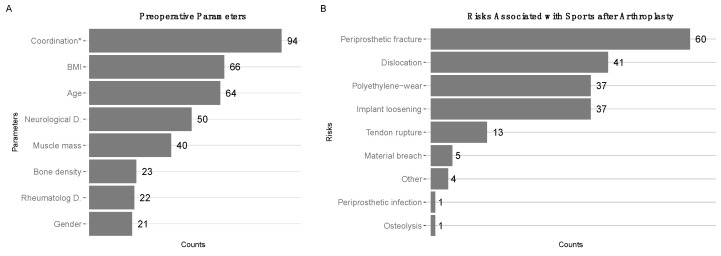
General patient evaluation and risk assessments: (**A**) Preoperative parameters ranked by estimated influence on return to sports (RTS) capacity of the patient. Coordination (i.e., preoperative experience with the type of sport) ranked highest with 94 counts, BMI (66 counts) second, age (64 counts) third. (**B**) Estimated risks associated with sports after arthroplasty: periprosthetic fractures (60 counts) ranked first, implant dislocation (41 counts) second, polyethylene wear and implant loosening (both 37 counts) ranked third.

Preoperative patient assessment: In total 78% of surgeons included assessment of the patient’s physical activity level in their standard preoperative patient work-up. According to the surgeons’ estimation, the most significant factors that influenced postoperative RTS capacity included coordination (i.e., previous experience in a specific type of sport) (94 counts), body mass index (BMI) (66 counts), and age (64 counts), less often neurological preconditions (50 counts) and muscle mass (40 counts) (Figure 1A).

Estimated risks associated with sports after arthroplasty: We asked surgeons to estimate the risks associated with sports after arthroplasty of the lower limb in general, among which “periprosthetic fractures” (60 counts) ranked first, while polyethylene wear (37 counts) and implant loosening (37 counts) ranked only third (Figure 1B).

### 3.3. Significance of Physical Activity after TKA

**Figure 2:**
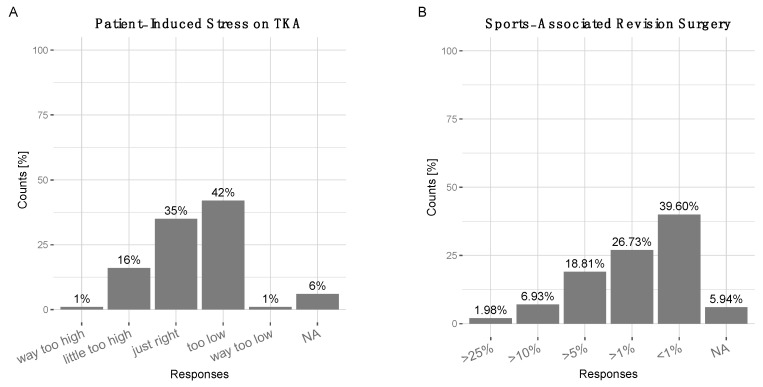
(**A**) Surgeons were asked to estimate the amount of patient-induced stress acting on the total knee arthroplasty. 1% ‘way too low’, 42% ‘too low’, 35 ‘just right’, 16% ‘a little too high’, 1 % ‘way too high, 6% ‘NA’. (**B**) Most surgeons estimated sports-associated revision surgery was ‘<1%’ (39.6%) or ‘>1%’ (26.73%), ‘>5%’ (18.81%), ‘>10%’ (6.93%), ‘>25%’ (1.98%).

There were 54.5% of surgeons who considered physical activity as important, 29.7% as very important—totaling 84.2% in favor of physical activity after knee arthroplasty. Only 8.9% considered it unimportant, 2% stated it did not matter, 5% NA. A total of 49% of surgeons did not think that RTS had a negative impact on the longevity of the knee implant, even so, 33% did (15% did not know, 4% NA). Conversely, 72.3% expected a positive impact of sports on the longevity of knee implant, only 14.9% did not, 8.9% did not know, 4% NA. A total of 42% of surgeons considered their patients’ weight bearing on the implant was “too low”, 1% “much too low”, while 35% considered it “just right”. A total of 16% stated that patient-induced weight bearing was “a little too high”, 1% “much too high” (6% NA) (Figure 2A). The data suggested that many experts (at least 43%) could encourage patients to increase physical activity overall. Surgeons estimated that the prevalence of sports-induced revision surgery after TKA was “<1%” (39.6%), “approximately 1%” (26.73%), “approximately 5%” (18.81%), “approximately 10%” (6.93%), and “above 25%” (1.98%) with 5.94% NA. (Figure 2B).

### 3.4. Surgical Decision-Making in Patients with High RTS Expectations after TKA

**Figure 3:**
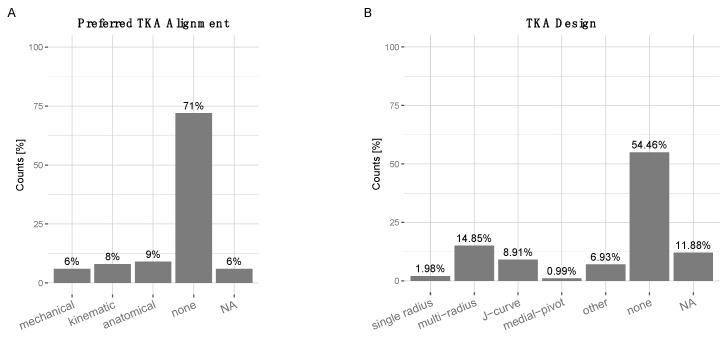
(**A**) Preferred TKA alignment in patients with high RTS expectations: 6% ‘mechanical’, 8% ‘kinematic’, 9% ‘anatomical’, 71% ‘no influence’, 6% NA. (**B**) Preferred total knee arthroplasty (TKA) design in patients with high RTS expectations. 1.98% ‘single radius’, 14.85% ‘multi-radius’, 8.91% ‘J-curve’, 0.99% ‘medial-pivot’, 6.93% ‘unspecified other’, 54.46% ‘no influence’, 11.88% NA.

**Figure 4:**
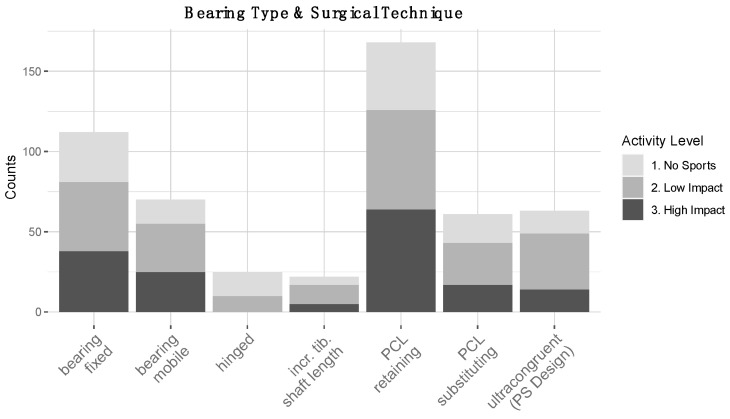
Preferred choice of bearing type and ligaments depending on expected patient activity level (multiple choices possible, absolute counts given): fixed bearings–‘high-impact’ (38 counts), ‘low-impact’ (43 counts), ‘no sports’ (31 counts). mobile bearing–‘high-impact’ (25 counts), ‘low-impact’ (30 counts), ‘no sports’ (15 counts). Hinged ‘high-impact’ (0 counts), ‘low-impact’ (10 counts), ‘no sports’ (15 counts). Increased tibial shaft length–‘high-impact’ (5 counts), ‘low-impact’ (12 counts), ‘no sports’ (5 counts). ‘PCL retaining’–‘high-impact’ (64 counts), ‘low-impact’ (62 counts), ‘no sports’ (42 counts). ‘PCL substituting’–‘high-impact’ (17 counts), ‘low-impact’ (26 counts), ‘no sports’ (18 counts). ‘ultra-congruent or PS design’–‘high-impact’ (14 counts), ‘low-impact’ (35 counts), ‘no sports’ (14 counts).

Surgical approach: 91% of surgeons stated high RTS expectations had no influence on the surgical approach, 3% stated they preferred “medial parapatellar”, 3% a “subvastus” approach 0% chose a “midvastus” approach, and 3% NA. TKA alignment: 71% said high RTS expectations in patients had no influence on their targeted TKA alignment, 9% preferred anatomical-, 8% kinematic-, 6% mechanical alignment, and 6% NA (Figure 3A). The majority 54.46% of surgeons stated that high expectations to RTS had no influence on their choice of TKA design. 1.98% “single radius”, 14.85% “multi-radius”, 8.91% “J-curve”, 0.99% “medial-pivot”, 6.93% “unspecified other”, 11.88% NA (Figure 3B). TKA bearing types and surgical techniques: In terms of surgical techniques, surgeons preferred fixed-bearings as well as PCL-retaining techniques for “high-impact”, “low-impact” as well as “no sports” (Figure 4).

### 3.5. Postoperative Treatment

**Figure 5:**
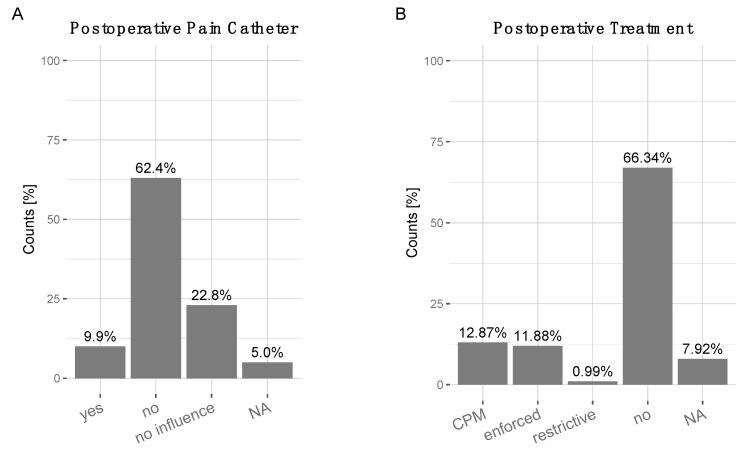
(**A**) Preference for intra-articular pain catheter application: 9.9% in favor (‘yes’), 61.4% not in favor (‘no’), 22.8% ‘no influence’, 5% NA. (**B**) Recommendation for postoperative treatment and rehabilitation: 12.87% ‘Continuous passive motion (CPM)’, 11.88% ‘enforced rehabilitation’, 0.99% ‘restrictive rehabilitation’, 66.34% no influence’, 7.92% NA.

Pain catheters: were applied by only 9.9% of surgeons. 62.4% did not opt for the application of any pain catheters, 22.8% stated it had no influence, 5% NA (Figure 5A). Postoperative treatment and rehabilitation: The majority of surgeons 66.34% did not recommend any specialized postoperative rehabilitation or treatment other than the standard rehabilitation programs in patients with high RTS expectations. At least 12.87% recommended a continuous passive motion (CPM), 11.88% an enforced rehabilitation program, 0.99% a more restrictive rehabilitation program and 7.92% NA (Figure 5B).

### 3.6. Recommendations for Patients with High RTS Expectations after TKA

**Figure 6:**
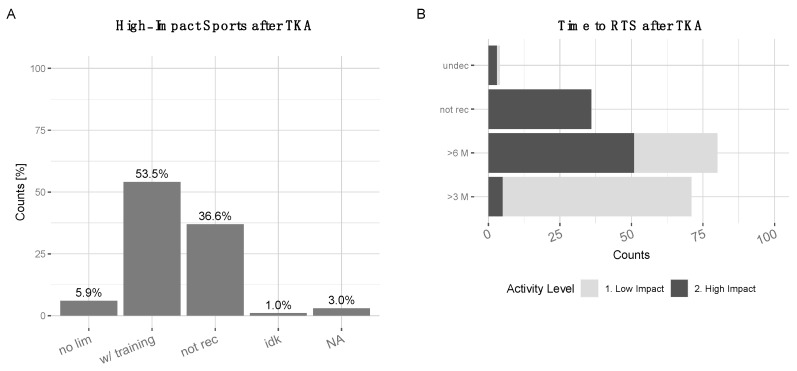
(**A**) Recommendations for high-impact sports after TKA: 5.9% ‘no lim’=‘no limitations’, 53.5% ‘w/ training’=‘with training’, 36.6% ‘not rec’=‘not recommended’, 1% ‘idk’=’I do not know’, 3% NA. (**B**) Time to return to sports (RTS) after TKA recommendations (absolute counts): High-impact sports (black)–‘>3M’ (5 counts), ‘>6M’ (49 counts), ‘not recommended’ (36 counts), ‘undecided’ (2 counts). Low-impact sports (light grey)–‘>3M’ (64 counts), ‘>6M’ (49 counts), ‘not rec’=‘not recommended’ (0 counts), ‘undec’=‘undecided’ (1 counts).

A total of 53.5% of surgeons recommended high-impact sports after TKA with adequate training of the patient, 36.6% did not recommend it at all. A total of 5.9% recommended high-impact sports without limitations. (1.0% did not know, 3% NA) (Figure 6A). Most low-impact sports were recommended after three months, while high-impact sports required at least six months of rehabilitation or rather it was not recommended at all (Figure 6B).

**Figure 7:**
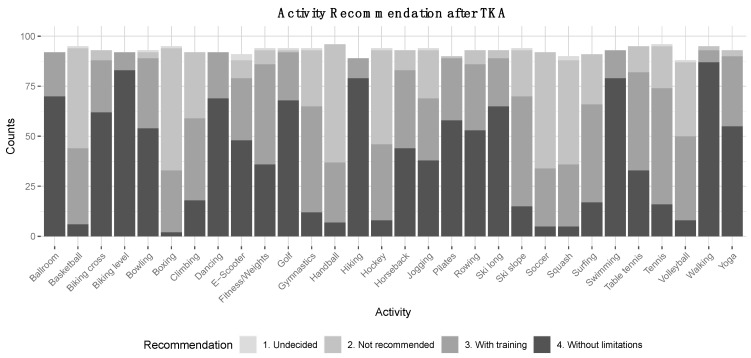
Activity recommendation after TKA. The y-axis represents absolute counts, the x-axis represents the type of activity/sport. ‘Ballroom’ has been abbreviated for ‘Ballroom dancing’. Recommendations ‘without limitations’ (dark grey), ‘with adequate training’ (medium grey), ‘not recommended’ (light grey), ‘undecided’ (lightest grey).

The results of this study showed that sports including basketball, boxing, gymnastics, handball, hockey, ski slope, soccer, squash, volleyball were recommended with greater restrictions compared to lower-impact sports, such as ballroom dancing, biking (both level and cross), dancing, golf, hiking, swimming, and walking. The lower-impact activity types were recommended without limitations (Figure 7).

## 4. Discussion

### 4.1. Sports after TKA

With 223 knee replacement surgeries per 100,000 capita, Germany ranks fourth after Austria, Luxembourg, and Finland in knee arthroplasties performed per annum [23]. Most surgeons, who participated in our survey, stated that preoperative experience with the type of sport was one of the most important factors for RTS in patients (Figure 1A). This was in line with previous observations [24,25], where patient-reported outcomes suggest a correlation between preoperative levels of physical activity and RTS capacity after TKA. Most patients return to low-impact sports after TKA with popular activities such as bicycle riding, swimming, hiking, and sometimes skiing (alpine) [26,27,28,29,30]. Previous similar studies observed that most surgeons are rather reluctant to recommend participation in high-impact sports after TKA [31,32], leading concerns were aseptic loosening due to increased wear and debris [33,34]. We observed that periprosthetic fractures were the leading concern in our study. This may be a reflection of an increased faith in improved implants; however, future investigations are needed to clarify this point.

The Clifford and Mallon classification was used to distinguish high-, intermediate-, and low-impact sports in previous surveys [21,31,32]. D’lima et al. were able to demonstrate in vivo knee forces during golfing, considered a low-impact sport, generated surprisingly high peak forces comparable to those found during jogging [35]. Interestingly, surgeons’ recommendations regarding jogging in our study were surprisingly liberal (38 recommended without limitations, 31 with prior training), while recommendations for tennis were much more restrictive (16 recommended without limitations, 58 with prior training), even though measured peak forces had been comparable to those found in jogging [35]. This was in line with previous similar studies, where tennis was not recommended [24]. Tennis constitutes abrupt stops and rapid changes of direction, whereas jogging is characterized by mostly continuous and rhythmic motions. Whether these differences between the two activities justify the varying recommendations remains to be seen. Further studies are needed to define relevant risk factors of a sport on the longevity and survivorship of modern TKAs.

The majority of surgeons who participated in this study responded that a return to low-impact sports was recommended after 3 months or 6 months (latest). Regarding high impact sports the responses were more controversial. Although available studies suggest that physical activity may negatively impact the longevity of the implant [26,33,34], other publications have proposed that at least moderate physical activity has no negative impact, instead, improving osteointegration and implant stability [36]. Indeed, higher wear rates have been observed in younger and more active patients [37]. Overall, the results of our survey suggest that according to surgeons’ opinions, a return to high-impact sports requires at least prior training and experience with the type of sport and a minimum of six months to return to it.

The question of return to high-impact sports after total joint replacement has been investigated previously [38,39,40] and studies that have been published on return to high-impact sports after TKA suggest encouraging results regarding short to intermediate outcomes [39,41]. However, whether the performance of high-impact sports reduces TKA implant longevity and survivorship requires longer investigation times of 20–30 years. Currently, return to high-impact sports may be reserved for a specific patient subpopulation.

**Figure A1:**
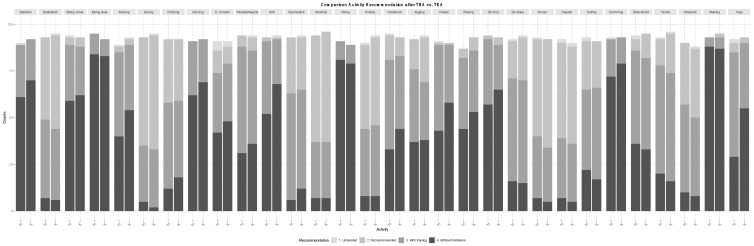
Comparison of activity recommendations after THA to TKA. H = Hip, K = Knee. Activity recommended without limitations (dark), with adequate training (grey), not recommended (light grey), undecided (lightest grey). Recommendations grouped hip vs. knee by activity in alphabetical order. ‘Ballroom’ refers to ballroom dancing.

When we compared our results with previously published recommendations for RTS after total hip arthroplasty (THA), we observed no striking differences between activity recommendations after THA vs. TKA (Figure A1). Knee arthroplasty implants and surgical techniques have a higher complexity compared to the adjacent hip joint, due to the anatomy and associated biomechanical bearing forces. Both joint replacement forms have developed significantly over the last 50 years. Interestingly, despite differences between the two joints, surgeons’ RTS recommendations were comparable and conformed to previously published data [32,42,43].

### 4.2. Surgical Techniques

Surgical approach: Surgical approaches in TKA include the traditional medial parapatellar [44], the subvastus surgical approach, characterized by quadriceps preservation [45] and midvastus surgical approach, a compromise between medial parapatellar and subvastus approach [46]. The surgical approaches have recently been reviewed in a Cochrane Systematic Review protocol [47]. The majority of surgeons responded that the surgical approach had no significant impact on patient outcome after TKA, in line with the current literature [48,49].

TKA alignment: The vast majority of surgeons (71%) opted for “no influence” of high RTS expectations on the targeted alignment. Early propositions claiming that clinical outcomes were dependent on good TKA alignment [50,51] have been refuted by more recent publications, which show that the different alignment options do not have a significant impact on clinical outcomes [52,53].

### 4.3. Implant Specifications

By increasing the postoperative range of motion (ROM) and mimicking the physiological motions of the native knee, present-day TKA designs aim to provide higher patient satisfaction and outcomes especially in younger and more active patients with high RTS expectations [7]. TKA design: Interestingly, the majority (54.5%) of surgeons opted for “no influence” on TKA design choices in patients with high RTS expectations. Some surgeons (14.1%) stated they preferred a multi-radius design in patients with high-RTS expectations. TKA design has undergone a tremendous evolution in the last 50 years [54]. Prominent designs such as the Attune Knee, which is a multi-radius trochlea groove design vs. the single radius trochlea groove PFC Sigma have shown excellent results in clinical studies [55]. The current literature provides inconsistent data regarding superiority of either design over the other [56,57,58,59,60,61]. This may be in line with the surgeons’ responses. Studies capable of demonstrating differences in effects may still be pending. Cruciate ligaments: The results of our survey showed that most surgeons opted for PCL-retaining surgical techniques, especially in patients with return to high-impact sports expectations. Cruciate ligaments are indeed crucial for the stabilization and ROM of the knee in three planes. The current literature is still controversial regarding this topic [62], most reviews suggest that no significant differences in outcome exist between PCL retaining and PCL sacrificing techniques [63,64,65,66,67]. However, it is important to note that these studies were not conducted in highly active patients. For the individual patient with high RTS expectations, minor increases in ROM of a few degrees or proprioception could make a meaningful difference [68]. Indeed, minor differences in proprioception in favor of the PCL retaining techniques were noted by some studies [65,69], suggesting that retaining the PCL may be important for functional capacity in some patients. Bearing type: The majority of surgeons favored a fixed bearing over a mobile bearing TKA in patients expecting to perform “high-” and “low-impact” as well as “no sports” after TKA. This suggested that the preferred choice of bearing type as well as ligament surgical techniques depended little on the expected patient activity level. Indeed, mobile bearings were initially introduced in TKA to cater towards expected higher functional demands of younger patients. Proposed advantages included reduced wear and consequent loosening of the implant [70]. The results have been reviewed by several authors and no advantages of mobile bearings could be confirmed thus far, instead, they were associated with complications and early-revision surgeries [71,72,73,74].

Postoperative training and rehabilitation are likely essential to acquire good ROM, function and RTS level. However, few studies demonstrate these effects in a systematic approach. Application of pain catheters: Most surgeons opted for “no application” of pain catheters after TKA in patients with high RTS expectations or no influence. There are various types of pain catheters available including intraarticular, continuous femoral nerve analgesia, epidural, and local infiltration analgesia (LIA) [75,76,77,78,79,80,81]. The rationale behind using pain catheters or application of a multimodal pain management is to promote early postoperative pain relief to allow quick recovery and rehabilitation. The current literature provides little evidence for significant differences in long-term outcome, in line with the surgeons’ assessments in our survey. Nevertheless, as pain is highly individual, the application, and associated risks, should be weighed on a case-by-case basis. Postoperative treatment: Surgeons were asked whether they recommended certain postoperative rehabilitation programs, such as continuous-passive motion (CPM), enforced rehabilitation, restrictive rehabilitation—65.7% of surgeons opted for no influence, 13.1% opted for CPM. The effects of CPM have been reviewed recently in a Cochrane Systematic Review [82], the authors found no significant advantages in clinical outcome. The lack of evidence regarding post-TKA rehabilitation may have prompted surgeons’ responses accordingly. However, as indication for surgical intervention knee arthroplasty broadens to encompass younger and more active patient clientele, the group of patients receiving knee arthroplasty is also becoming more heterogeneous. Previous clinical outcome measurements may not be able to differentiate between the groups and standard post-operative rehabilitation program may not meet the needs of every patient. As recently demonstrated, goal attainment scaling (GAS) may be one approach to improve satisfaction for active patients with high RTS expectations [29,83].

In order to increase patient satisfaction and physical activity as well as improving long-term knee arthroplasty outcomes, new approaches for implant designs as well as surgical techniques have been developed over the years. These various alterations have led to a wide spectrum of available techniques and improved implant materials that are hypothesized to increase implant longevity and weight-bearing capacities [52,53,84]. However, knee arthroplasties cannot replicate the biomechanical capabilities of the healthy knee and present with their own limitations and the improvement of implants may not always translate to improved functions in patients. On the other hand, benefits to only a small, highly active subpopulation may be valuable but remain undetected in larger reviews and meta-analyses.

### 4.4. Limitations

This study has several limitations. It was designed to assess expert opinions of arthroplasty experts for patients with high RTS expectations and an active lifestyle after TKA. This study does not provide in vivo tested evidence and the results may be subjected to the surgeons’ observer-bias as well as recall-bias: Patients often return to low-impact sports after TKA [85]. A participation in high-impact sports is still rare and relates to only a very specific sub-population of TKA patients [27]. Most surgeons responded that their patients’ level of physical activity could be encouraged and that observed sports-associated complications after TKA were rather low. This observation may falsely encourage the impression that patients with TKAs can safely return to sports, including higher-impact types, although the arthroplasties have not yet been put that type of test. Another limitation may be that surgeons observed different patient age groups with different types of demands. This may lead to their perception of sports after TKA to be skewed. It is important to note that available in vivo evidence measuring load bearing forces and wear in patients who received knee arthroplasty, do not immediately transfer to patients with an active lifestyle and high RTS expectations. To the best of our knowledge, no such studies exist.

## 5. Conclusions

Knee arthroplasty surgery in young and active patients with high RTS expectations make up a growing subgroup of patients with TKA. Evidence regarding the best standard-of-care for patients receiving knee arthroplasty with high RTS expectations is scarce and available findings are often controversial [68]. Biomechanical studies designed to test load bearing and force distributions in patients with knee arthroplasty do not allow a direct translation to the functional limitations in the active patient [86,87,88,89]. As a consequence, most recommendations and patient counselling are based on surgeons’ and physicians’ individual experience and training. There seems to be a general agreement that patients should be counselled toward an active lifestyle, despite observed higher wear rates and debris in younger and more active patients, concerns may need to be weighed against obesity and secondary effects of inactivity [33,90]. In addition, recommendations are often dependent on patients’ preoperative abilities and coordination and often result in a return to low impact sports. High-impact sports are recommended by some, which may reflect an increased faith in improved implants, as no available data exists demonstrating the long-term survivorship of these implants in highly active patients. In addition, improved specific outcome measurements may be needed to capture specific needs of this patient cohort.

## Supplementary Materials

The following are available online at https://www.mdpi.com/2077-0383/10/1/54/s1, S1: The original questionnaire; S2: The English translation of the original questionnaire; S3: Here we provide the full R data frame encoding all questionnaire items and responses.

## Abbreviations

AA	anatomical alignment
CAS	computer-assisted surgery
GAS	goal attainment scaling
KA	kinematic alignment
LIA	local infiltration analgesia
MA	mechanical alignment NA
NA	not available, no response
PCR	posterior-cruciate ligament retaining
PCS	posterior-cruciate ligament substituting/sacrificing
PS	posterior stabilized (knee arthroplasty)
PSI	patient-specific instrumentation
RAS	robot-assisted surgery
ROM	range of motion
THA	total hip arthroplasty
TKA	total knee arthroplasty
UID	unique identity
