# Supplementary material for: Recommendations for Patients with High Return to Sports Expectations after TKA Remain Controversial
Source: J Clin Med. 2020 Dec 26;10(1):54. doi: 10.3390/jcm10010054 (PMC7796219; doi:10.3390/jcm10010054)
Supplement: Supplementary file 1 [file jcm-10-00054-s001.zip › S1_AE_Questionnaire_RTS_TKA_DE.pdf]

DEUTSCHE GESELLSCHAFT FÜR ENDOPROTHETIK

EINE UMFRAGE DER AE - DEUTSCHE GESELLSCHAFT FÜR ENDOPROTHETIK E.V. ZUR EINSCHÄTZUNG DER SPORTLICHEN BELASTUNGSFÄHIGKEIT VON PATIENTEN NACH GELENKIMPLANTATIONEN

– Wir danken Ihnen für Ihre geschätzte Teilnahme!

O. Freiwillige Angaben

Name:____ Alter: ___________

Tätigkeitsort: ____

1 Allgemeine Fragen

1.1 In welchem Jahr ca. haben Sie ihre Approbation erhalten?

O vor 1980
O 1980 -1990
O 1990 - 2000
O 2000 - 2010
O 2010 – 2020

1.2 Wie hoch schätzen Sie ihre eigene sportliche Aktivität ein?

O sehr hoch, ich mache täglich Sport
O hoch, ich mache mehrmals die Woche Sport
O mittel, ich mache ab und zu Sport
O niedrig, ich mache gar kein Sport

1.3 Gehört die Frage nach der sportlichen Aktivität des Patienten vor Endoprothesenersatz zu Ihrem Standardfragekatalog?

O Ja
O Nein
O Weiß nicht
O egal

1.4 Welche **präoperativen Parameter** sind für Sie hinsichtlich der postoperativen Sportfähigkeit in der Beurteilung relevant?

O Alter
O Geschlecht
O BMI
O Muskelmasse
O Koordination/Ausübung der Sportart vor der geplanten Operation
O Knochendichte
O neurologische Nebenerkrankungen
O rheumatologische Grunderkrankungen

1.5 Welches ist Ihrer Einschätzung nach das **Hauptrisiko** durch Sport nach Implantation einer Endoprothese?

(maximal 2 Nennungen)

O Luxation
O Periprothetische Fraktur
O vorzeitige Prothesenlockerung
O PE-Abrieb
O Periprothetische Infektion
O Osteolyse
O Materialbruch
O Bandruptur
O Andere _____________________________ (welche?)

3 Spezielle Fragen zum Gelenkersatz am Knie

3.1 Für wie wichtig halten Sie sportliche Aktivität nach **Knieendoprothesenersatz**?
- O sehr wichtig
- O wichtig
- O nicht wichtig
- O egal

3.2 Glauben Sie, dass sportliche Aktivität die Haltbarkeit einer **Knieendoprothese** <u>negativ</u> beeinflusst (Standzeit reduziert)?
- O Ja
- O Nein
- O weiß ich nicht
- O egal

3.3 Glauben Sie, dass die sportliche Aktivität die Funktion der **Knieendoprothese** <u>positiv</u> beeinflusst?
- O Ja
- O Nein
- O weiß ich nicht
- O egal

3.4 Die **Belastung**, welcher sich Patienten mit einer **Kniendoprothese** aussetzen ist in der Regel
- O Viel zu hoch
- O Etwas zu hoch
- O Genau richtig
- O Zu niedrig
- O Viel zu niedrig

3.5 Wie führen Sie die **Fehlschläge** von **Knieendoprothesen** auf Überlastung bzw. Sport zurück?
- O In mehr als 50% der Fälle
- O In mehr als 25% der Fälle
- O In mehr als 10% der Fälle
- O In mehr als 5% der Fälle
- O In mehr als 1% der Fälle
- O In weniger als 1% der Fälle

3.6 Hat ein hoher sportlicher Anspruch Einfluss auf die Wahl ihres **operativen Zugangsweges**?
- O Ja,
  wenn ja, welchen würden sie in diesem Fall nutzen?
    - O medial parapatellär
    - O subvastus
    - O midvastus
- O Nein

3.7 Hat ein hoher sportlicher Anspruch Einfluss auf das **angestrebte Alignment**?
- O Ja,
  wenn ja, welchen würden sie in diesem Fall nutzen?
    - O Mechanisches Alignment
    - O Kinematisches Alignment
    - O Anatomisches Alignement Residueller Varus- bzw. Valgus
- O Nein

3.8 Würden Sie bei möglicher Indikation einer **unikondylären Schlittenprothese** mit hohem Sportanspruch zu einer **bikondylären Endoprothese** aufgrund der gewünschten Belastung wechseln?

- O Ja
- O Nein

3.9 Glauben Sie, dass das Design Ihrer Knie-Endoprothese Einfluss auf die Sportfähigkeit des Patienten hat?

- O Ja,
  Wenn ja, welches Design empfehlen Sie bei hohem sportlichem Anspruch?
    - O single-radius
    - O multi-radius
    - O J-Curve
    - O medial-pivot
    - O andere__________________(welches Design?)
- O Nein

3.10 Welchen **Kopplungstyp für eine Knie-TEP** empfehlen Sie bei den gewünschten sportlichen Aktivitäten des Patienten?

Kopplungstyp	High-Impact*	Low-Impact*	Kein Sport
HKB erhaltend	o	o	o
HKB ersetzend	o	o	o
Ultrakongruent (PS Design)	o	o	o
Fixed Bearing	o	o	o
Mobile Bearing	o	o	o
Tibiale Stielverlängerung	o	o	o
Gekoppelte Prothese	o	o	o

3.11 Würden Sie bei hohem Sportanspruch zur **Anlage von Schmerzkathetern** (N.femoralis, N. ischiadicus) raten?
O Ja,
O Nein
O Egal

3.12 Hat ein hoher sportlicher Anspruch Einfluss auf die **Nachbehandlung**?
O Ja,
wenn ja, welche würden sie in diesem Fall empfehlen?
O Nutzung einer CPM (continuous passive motion) Schiene
O forcierte Nachbehandlung
O restriktivere Nachbehandlung
O Nein

3.13 Ihr Patient äußert den Wunsch nach der **Knie-TEP OP** zu zuvor ausgeübten **high-impact*** Sportarten zurückzukehren. Welche Aussage treffen Sie?
O Ich empfehle es uneingeschränkt nach einer gewissen Schonzeit
O Ich empfehle es nur bei adäquater Schulung des Patienten über die Bewegungen/Risiken
O Ich empfehle keine high-impact Sportarten nach Gelenkersatz
O Ich weiß es nicht

3.14 Wie lautet Ihre **Sport Empfehlung** nach Implantation einer **Knie-Totalendoprothese (Knie-TEP)?**

Sportart	n. 3 Monaten	n. 6 Monaten	Nicht empfohlen	Unentschieden
**High-Impact***	O	O	O	O
**Low-Impact***	O	O	O	O

[1]

[1] *Zu den Low-Impact Sportarten zählen u.a. Schwimmen, Gehen, Pilates und elliptisches Kardiotraining (Crosstrainer). Sie zeichnen sich durch sanfte und flüssige Bewegungen aus, bei denen das Verletzungsrisiko gering ist. Zu den High-Impact Sportarten zählen u.a. Laufen, Skifahren, Squash, Basketball und Boxen, bei denen das Verletzungsrisiko hoch ist, insbesondere, wenn Patienten nicht trainiert oder ungeschult sind.

5. Wie lautet Ihre Empfehlung für Patienten nach **Knie-Total-Endoprothesenersatz**?

Sportart	uneingeschränkt empfohlen	nur mit Schulung	nicht empfohlen	unentschieden
Basketball	O	O	O	O
Bowling	O	O	O	O
Boxing/Kampfsport	O	O	O	O
E-Scooter fahren	O	O	O	O
Fitness/Gewichte Heben	O	O	O	O
Fußball	O	O	O	O
Gesellschaftstanz (Partner)	O	O	O	O
Golf	O	O	O	O
Handball	O	O	O	O
Hockey	O	O	O	O
Joggen	O	O	O	O
Klettern	O	O	O	O
Pilates	O	O	O	O
Radfahren Ebene	O	O	O	O
Radfahren Unebene	O	O	O	O
Reiten	O	O	O	O
Rudern	O	O	O	O
Schwimmen	O	O	O	O
Skifahren (alpin)	O	O	O	O
Skilanglauf	O	O	O	O
Spazieren	O	O	O	O
Squash	O	O	O	O
Surfen	O	O	O	O
Tanzen	O	O	O	O
Tennis	O	O	O	O
Tischtennis	O	O	O	O
Turnen	O	O	O	O
Volleyball	O	O	O	O
Wandern	O	O	O	O
Yoga	O	O	O	O
