# Supplementary material for: Recommendations for Patients with High Return to Sports Expectations after TKA Remain Controversial
Source: J Clin Med. 2020 Dec 26;10(1):54. doi: 10.3390/jcm10010054 (PMC7796219; doi:10.3390/jcm10010054)
Supplement: Supplementary file 1 [file jcm-10-00054-s001.zip › S2_AE_Questionnaire_RTS_TKA_ENG.pdf]

DEUTSCHE GESELLSCHAFT FÜR ENDOPROTHETIK

A SURVEY OF THE AE – GERMAN SOCIETY FOR ARTHROPLASTY E.V. TO ASSESS CURRENT RECOMMENDATIONS FOR RETURN TO SPORT AFTER JOINT REPLACEMENT

Please return your completed survey form at the registration or via mail to
Dr. med. PhD Tu-Lan Vu-Han Centrum für Muskuloskeletale Chirurgie (CMSC) | Charité – Universitätsmedizin Berlin, Charitéplatz 1 | 10117 Berlin | Tel. +49 30 450 615 145, E-Mail: tu-lan.vu-han@charite.de
– We thank you for your participation!

O. Voluntary Information

Name:____ Age: ___________

Place of practice:

____

1 General Questions

1.1 "In which year did you receive your Approbation?"
- O vor 1980
- O 1980 -1990
- O 1990 - 2000
- O 2000 - 2010
- O 2010 – 2020

1.2 "How high do you rate your own athletic activity?"
- O "very high, I do sports daily"
- O "high, I do sports multiple times a week"O mittel, ich mache ab und zu Sport
- O "medium, I do sports every now and then"
- O "low, I do not do sports"

1.3 "Is the question of the patient's sporting activity before joint replacement part of your standard patient interview?"
- O „Yes"
- O "No"
- O "I do not know"
- O "It does not matter"

1.4 "Which preoperative parameters are relevant for you with regard to postoperative sporting ability in the assessment?"
- O „Age"
- O „Gender"
- O „BMI"
- O „Muscle mass"
- O "Coordination/Experience of the sport before planned surgery"
- O "Bone density"
- O "Neurologic precondition "
- O "Rheumatologic precondition"

1.5 "What do you think is the **main** risk from sport after implantation of an endoprosthesis? (Maximum 2 answers)"
- O "Joint dislocation"
- O "Periprosthetic fracture"
- O "Implant loosening"
- O "Polyethylene-wear"
- O "Periprosthetic infection"
- O "Osteolysis"
- O "Material breach"
- O "Tendon rupture"
- O „Other ______________________________ (which?)"

3 Specific questions to knee arthroplasty

3.1 "How important do you think sporting activity after knee replacement surgery?"
- O "very important"
- O "important"
- O "not important"
- O "does not matter"

3.2 "Do you think that physical activity has a **negative** impact on the durability of a knee arthroplasty (reduced lifespan)?"
- O „Yes“
- O „No“
- O „Do not know”
- O „Does not matter”

3.3 "Do you think that physical activity has a **positive** impact on the durability of a hip arthroplasty (reduced lifespan)?"
- O „Yes“
- O „No“
- O „Do not know”
- O „Does not matter”

3.4 "The burden that patients with a **knee arthroplasty** expose themselves to is usually ..."
- O "Way too high"
- O "A little too high"
- O "Just right"
- O "Too low"
- O "Way too low"

3.5 "How often do you attribute failure of **hip arthroplasty** to stress overload or sports?"
- O "In more than 50% of revision surgeries"
- O "In more than 25% of revision surgeries"
- O "In more than 10% of revision surgeries"
- O "In more than 5% of revision surgeries"
- O "In more than 1% of revision surgeries"
- O "In less than 1% of revision surgeries"

3.6 "Does a high athletic standard influence the choice of your surgical access route? If yes, which one do you choose?"
- O Yes,
  if yes, which surgical approach do you prefer?
  - O medial parapatellar
  - O subvastus
  - O midvastus
- O No

3.7 "Does a high level of sporting ability influence the desired alignment? If yes, which one?"
- O Yes,
  if yes, which alignment do you prefer?
  - O "mechanical alignment"
  - O "kinematic alignment"
  - O "anatomical alignment, residual varus- or valgus"
- O No

3.8 "Do you prefer a unicondylar sled prosthesis to a bicondylar one in case of high physical demands?"

- O Yes
- O No

3.9 "Does the design of a knee arthroplasty affect athletic ability? If so, which design do you choose?"

- O Yes,
  If yes, which design do you prefer for patients with high return to sports expectations?
  - O Single-Radius
  - O Multi-Radius
  - O J-Curve
  - O Medial-Pivot
  - O andere__________________(which design?)
- O No

3.10 "What type of coupling for a TKA do you recommend for the patient's desired sporting activities?"

Kopplungstyp	High-Impact*	Low-Impact*	No Sport
posterior cruciate retaining (PCR)	O	O	O
posterior cruciate substituting (PCS)	O	O	O
Ultracongruent (posterior stabilizing design)	O	O	O
Fixed Bearing	O	O	O
Mobile Bearing	O	O	O
Increased tibial shaft length	O	O	O
Hinged prosthesis	O	O	O

3.11 "Would you advise to apply pain catheters (N. femoralis, N. sciatic nerve) in patients with high athletic activity expectations?"

O „Yes“,
O “No”
O “Does not matter”

3.12 "Does a high athletic standard influence post-treatment (NB)?"

O Yes,
wenn ja, welche würden sie in diesem Fall empfehlen?
- O "Yes, application of a continuous passive motion treatment"
- O "Yes, enforced rehabilitation"
- O "Yes, restrictive rehabilitation"

O No

3.13 "Your patient expresses the wish to return to previously practiced high-impact sports after the knee arthroplasty surgery. Which statement do you make?"

O "I recommend it without limitations after a certain time of recovery”
O "I recommend it only after adequate training of the patient in regard to movements and risks."
O "I recommend no high-impact sports after arthroplasty"
O "I leave it to the patient and make no specific statement."

3.14 "What is your recommendation for sports after a **total knee arthroplasty (TKA)?"**

Type of sport	"after 3 months"	"after 6 months"	"not recommended"	"undecided"
High-Impact*	O	O	O	O
Low-Impact*	O	O	O	O

[1]

[1] *Low impact sports include swimming, walking, pilates, elliptical training. General characteristics are smooth and even body movements. In contrast, high-impact sports include running, skiing, squash, basketball and boxing. They generally have an increased risk for injury due to abrupt movements.

5. “What is your recommendation for patients after **total knee arthroplasty**?"

Sportart	without limitation	with training	not recommended	undecided
“Basketball”	O	O	O	O
“Bowling”	O	O	O	O
"Boxing/Martial Arts"	O	O	O	O
"E-Scooter"	O	O	O	O
"Fitness/Weights"	O	O	O	O
"Soccer"	O	O	O	O
"Ballroom Dancing"	O	O	O	O
"Golf"	O	O	O	O
"Handball"	O	O	O	O
"Hockey"	O	O	O	O
"Jogging"	O	O	O	O
"Climbing"	O	O	O	O
"Pilates"	O	O	O	O
"Biking level"	O	O	O	O
"Biking cross"	O	O	O	O
"Horseback riding"	O	O	O	O
"Rowing"	O	O	O	O
"Swimming"	O	O	O	O
"Ski slope"	O	O	O	O
"Ski long"	O	O	O	O
"Walking"	O	O	O	O
“Squash”	O	O	O	O
"Surfing"	O	O	O	O
“Dancing"	O	O	O	O
"Tennis"	O	O	O	O
"Table tennis"	O	O	O	O
"Gymnastics"	O	O	O	O
“Volleyball”	O	O	O	O
“Hiking”	O	O	O	O
“Yoga”	O	O	O	O

Please return your questionnaire at the reception or via e-mail to
Dr. med. Tu-Lan Vu-Han, PhD Centrum für Muskuloskeletale Chirurgie (CMSC) | Charité – Universitätsmedizin Berlin, Charitéplatz 1 | 10117 Berlin | Tel. +49 30 450 615 145, E-Mail: tu-lan.vu-han@charite.de
– We thank you for your participation!
